# Sedation versus protective stabilization for dental treatment of children with caries and challenging behavior at the dentist (CHOOSE): a study protocol for a non-randomized clinical trial

**DOI:** 10.1186/s12903-021-01594-0

**Published:** 2021-05-12

**Authors:** Gabriela Seabra da Silva, Anna Alice Anabuki, Karolline Alves Viana, Patricia Corrêa-Faria, Mônica Maia Moterane, Tamara Kerber Tedesco, Paulo Sucasas Costa, Marie Therese Hosey, Daniela Prócida Raggio, Luciane Rezende Costa, CEDACORE Collaborative Group

**Affiliations:** 1 Department of Pediatric Dentistry, School of Dentistry, University of São Paulo, São Paulo, Brazil; 2 Dentistry Graduate Program, Universidade Federal de Goias, Goiânia, GO Brazil; 3 Graduate Program in Dentistry, Ibirapuera University, São Paulo, Brazil; 4 Faculty of Medicine, Universidade Federal de Goias, Goiânia, GO Brazil; 5 Pediatric Dentistry, Centre for Oral Clinical and Translational Sciences, Faculty of Dentistry, Oral and Craniofacial Sciences, King’s College London, London, UK; 6 Faculty of Dentistry, Universidade Federal de Goias, Primeira Avenida, s/n, Goiânia, GO CEP 74605-220 Brazil

**Keywords:** Dental anxiety, Dental care for children, Dental caries, Conscious sedation, Physical restraint, Child behavior, Cost-effectiveness analysis

## Abstract

**Background:**

There is a lack of evidence on the effectiveness of moderate sedation in pediatric dentistry, compared to protective stabilization, which remains routinely used in Brazil despite moral questions. This prospective non-randomized clinical trial's objective is to evaluate the effectiveness of moderate sedation, compared to the protective stabilization, in the dental care of children with dental behavior management problems.

**Methods:**

Participants will be 152 children under seven years of age with early childhood caries (ECC) who need specialized dental treatment due to a history of challenging behavior during dental care. The interventions to be compared are moderate sedation with oral administration of ketamine and midazolam and protective stabilization. The primary endpoint will be the child's behavior during treatment assessed using the Ohio State University Behavioral Rating Scale (OSUBRS). The secondary outcomes are (A) child's – behavior according to the visual analogue scale, anxiety, pain, and physiological stress; (B) parent's – satisfaction and anxiety; (C) family and child – impact on oral health-related quality of life (OHRQoL); (D) dentist's – satisfaction and stress; (E) procedure – adverse events of the intervention and dental treatment longevity. A cost-effectiveness analysis will be performed from the perspective of the Brazilian Unified Health System (SUS).

**Discussion:**

Considering the primary outcome, this study hypothesis is that sedated children have better behavior during dental treatment than children whose behavior was managed by protective stabilization without sedation. Additionally, at the end of 12 months, we expect to identify participants' reported outcomes and objective measures related to dental behavior in early childhood.

*Trial registration* Clinicaltrials.gov registration NCT04119180 on October 8th, 2019. https://clinicaltrials.gov/ct2/show/NCT04119180

**Supplementary Information:**

The online version contains supplementary material available at 10.1186/s12903-021-01594-0.

## Background

Proper management of fear/anxiety (DFA) and behavioral problems during dental treatment (DBMP) are essential aspects of a humanized philosophy in caring for the child. The history of negative experience with dental treatment and age below five years of age are related to non-collaborative behavior in future consultations [1].

At least one child in ten presents some DFA degree that prevents his/her ability to tolerate dental treatment [2]. Specifically, in Brazil, dental fear can affect 21.6% of Brazilian children [3]. Advanced behavioral management methods for children with dental treatment needs and DFA or DBMP are sedation, protective stabilization, and general anesthesia [4].

Dental sedation benefits patients with dental fear and anxiety, minimizing pain and physical discomfort during treatment [5]. Sedation can be minimal, moderate, or profound, depending on the level of depression of consciousness that the patient reaches. In moderate sedation, there is medication-induced depression of consciousness; patients respond to verbal commands, alone or accompanied by light tactile stimulation, and maintained cardiovascular function, with no need for intervention to support the airways [6]. The efficacy analysis of such advanced methods (except for protective stabilization alone) was the subject of two Cochrane systematic reviews [5, 7]. One concluded that there is some weak evidence that oral midazolam is an effective sedative agent for children undergoing dental treatment and that there is weak evidence that inhalation of nitrous oxide can also be effective [5]. The other indicated no studies that allowed comparing the efficacy of sedation versus general anesthesia for dental treatment to children and adolescents under 18 years of age [7]. The effect of behavioral management techniques in pediatric dentistry remains questionable [8], even when it comes to non-pharmacological strategies [9, 10].

In this sense, protective stabilization is a chapter apart. While it has its routine use in Brazil [11], in the United Kingdom, the so-called Clinical Holding should only be used when there is an immediate need to perform a procedure on the child, provided that the person responsible consents and, if possible, the child agrees [12]. In the United States, protective stabilization is restricted to urgent cases in which there is no alternative behavioral management option [13]. Discussions in the Brazilian context indicate the ethical problems related to protective stabilization in pediatric dentistry. These concerns include lack of professionals’ training, failure to analyze risks and benefits, inconsistent indication, use in a wide variety of situations and non-urgent procedures, inattention with the caregivers' opinion, and inattention to the child’s autonomy [14].

However, to the best of our knowledge, no clinical trials comparing the use of sedation to protective stabilization in pediatric dentistry are available to date. This theme is essential for developing pediatric practice in Brazil and has a global impact since protective stabilization is still considered necessary for many countries. This prospective non-randomized clinical trial's objective is to evaluate the effectiveness of the use of moderate sedation, compared to protective stabilization, in the dental care of children with fear, anxiety, or behavior problems and identify associated factors.

## Methods

### Trial design

According to the guideline Standard Protocol Items: Recommendations for Interventional Trials (SPIRIT), the present protocol was reported, as detailed in Additional file 1.

This investigation is a non-randomized, superiority, comparative, parallel, two-arm clinical trial (sedation and protective stabilization), with 1:1 allocation. The study is non-randomized due to sedation being available only in one of the centers. Similarly, it will not be possible to mask the interventions as they are vastly different.

### Ethical considerations

The protocol was approved by the local ethics committee from the Federal University of Goias (CAAE #14585219.5.0000.5083) and the School of Dentistry of the University of Sao Paulo (CAAE #14585219.5.3001.0075). It was recorded in the database for registration of clinical studies (Clinicaltrials.gov registration NCT04119180). Participants will be included after their legal guardians have signed an informed consent form containing detailed information about the research, and the children nod their participation. Because the studied population will be children with challenging behavior, their informed assent will be waived.

### Study setting

The study will take place in outpatient clinics of two dental schools that have Know-how about this research:

- Federal University of Goiás (UFG): this institution conducts sedation of children and adults for dental treatment, following international guidelines and providing adequate infrastructure.
- University of Sao Paulo (USP): in this institution, the advanced method "protective stabilization" is performed.

Most patients referred to both institutions are from the Brazilian National Health System (SUS).

### Participants eligibility

The study population is children two years to under seven years of age with dental caries, who need specialized dental treatment due to the history of challenging behavior (behavior management problem) with dental care, according to the criteria:

#### Inclusion

- Children presenting cavities that need dental restoration among other dental treatment needs;
- Physical status ASA I (healthy) or II (mild and controlled systemic disease—persistent asthma, for example) children [6];
- Medical history without neurological or cognitive impairment;
- Children who do not use medicines that may impair cognitive functions;
- Children at low risk for airway obstruction (Mallampati less than two or tonsil hypertrophy occupying less than 50% of the oropharynx) [15].

#### Exclusion

- Children with positive or definitely positive behavior [16] in the dental examination session;
- Non-attendance to the first intervention appointment after three scheduling attempts;
- Chronic use of systemic corticosteroids because they influence the salivary cortisol that will represent physiological stress.

### Interventions

#### Moderate sedation

To be performed in NESO/UFG, with the association of midazolam (0.5 mg/kg, maximum 20 mg) and ketamine (4.0 mg/kg, maximum 100 mg) to be administered orally by a physician in the drug administration room. The leading accompanying adult should sit in the dental chair along with the child throughout the treatment.

#### Protective stabilization

To be performed at USP, after informed consent from the parents. The leading accompanying adult should sit in the dental chair together with the child and restraint movements of legs and arms when needed. A dental assistant supports the child's head contained during the session.

### Changes

The intervention with sedation may change according to the need of some participant in the following situations:

- When the child does not ingest oral medication, the drug association will be administered intranasally with an atomizer (ketamine 4.0 mg/kg, maximum 100 mg + midazolam 0.2 mg/kg, maximum 5 mg).
- When the child moves and jeopardizes his safety or treatment quality, he will receive protective stabilization in the sedation group.
- If the accompanying adult does not want to witness dental care, this will run as planned.
- If the child is suffering an adverse event from the drug at the first session or presenting challenging behavior throughout the session, the sedative regimen may be altered in the next appointment at the physician's discretion, or the child may be referred to general anesthesia instead.
- At the request of the legal guardian, if the latter is dissatisfied with the procedure.

Children who receive protective stabilization may also have their care suspended at the legal guardian's request or when their behavior jeopardizes their safety or the quality of treatment.

Regardless of any decision to modify or interrupt the intervention assigned, participants will be kept in the study whenever possible to collect monitoring data and avoid information loss.

### Adherence

The stimulus to the participant's adherence to the treatment and follow-up sessions will happen through contacts via mobile and social networks, humanized care, and clear explanations about the importance of their participation for their benefit. Losses are defined as non-attendance to follow-up consultations after three scheduling attempts.

### Concomitant care

The professionals will apply non-pharmacological behavioral management techniques in both groups according to the child's age and need, such as positive reinforcement and descriptive praise, ask-speak-ask, tell-show-do, non-verbal communication, memory restructuring, direct observation/modeling, distraction [4]. Assertive voice control (can be potentially aversive) will not be recommended; associated use of nitrous oxide will not be allowed.

### First consultation and intervention consultation

Certified pediatric dentists will perform the standardized dental examination, professional prophylaxis, and physical examination in both centers. At the same time, an assistant will note the diagnosis and the need for treatment (as detailed in the Additional file 2). During this procedure, non-pharmacological behavioral management methods will be used as necessary.

If the child presents negative/definitively negative behavior [16] in this consultation, he/she will be directly scheduled for the intervention visit. If a child has positive/definitely positive behavior during the examination, the pediatric dentist will restore one tooth (by using the Atraumatic Restorative Treatment restorative technique) to confirm the child's behavior in the face of more invasive stimuli. If positive behavior continues, the child will be excluded from the research but will continue to be routinely treated by the team. If the child demonstrates negative behavior, the child will be scheduled for the intervention visit with sedation (center UFG) or protective stabilization (center USP).

**Fig. 1:**
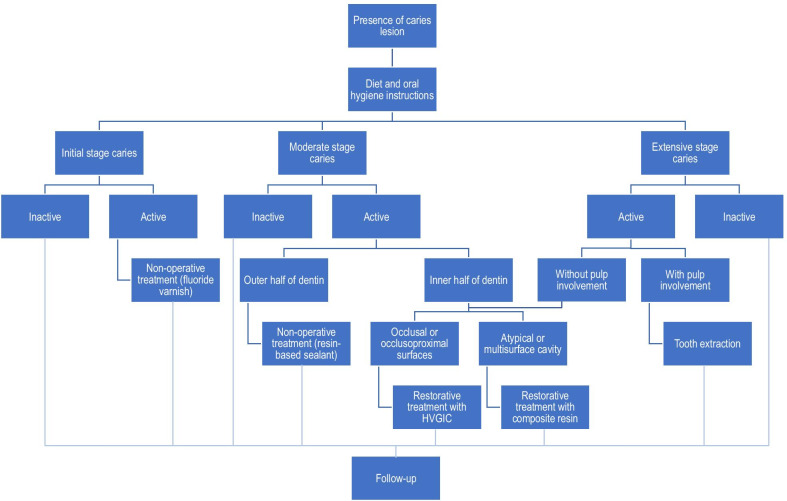
Organization chart of the decision-making process of teeth included in the trial

The child will have his treatment completed in as many consultations as necessary, using sedation (UFG) or protective stabilization (USP). When the child concludes the treatment, he/she will be scheduled for return in four months. Figure 1 displays the decision-making process and indicated treatments according to the diagnosis.

### Follow-up visits

The child should return to the UFG or USP centers at four, eight, and 12 months for follow-up. If any need is noticed in the follow-up, one trained operator will perform the treatments. Oral hygiene instructions, diet sugar control, and fluoride use will be repeated every return visit for all children.

### Outcomes

The primary outcome is the child's behavior, which will be assessed using the Ohio State University Behavioral Rating Scale (OSUBRS) in the digital videos recorded during the intervention [17]. The analysis of the videos will be performed by trained and calibrated observers with the aid of the Observer XT software (Noldus Information Technology, The Netherlands), which allows evaluating the behavior in total during each session, obtaining the percentage of scores, being: 1 – quiet, 2 – cry without movement, 3 – movement without crying, and 4 – struggling. Measurements for each group will be synthesized as mean (or median) and standard deviation (or quartiles).

The secondary outcomes are related to children's, families, and dentists' reports and other objective measures from the dental care (Table 1).

**Table 1:** Secondary outcomes and their respective analysis metrics

Secondary outcome	Participant level analysis metrics	Aggregation method	Measurement time
Child behavior, according to the dentist	10 cm Visual Analogue Scale (VAS)	Scores (ordinal scales)	Per session: First consultation, treatment sessions, follow-up sessions of 4, 8, and 12 months
Child anxiety	Facial Image Scale (FIS)	Scores (ordinal scale)	Per session: First consultation, treatment sessions, follow-up sessions of 4, 8, and 12 months
Oral health-related quality of life	The Brazilian version of Early Childhood Oral Health Impact Scale (B-ECOHIS) [18]	Scores (ordinal scale)	Per session: First consultation, follow-up sessions of 4, 8, and 12 months
Parents' satisfaction and anxiety	10 cm VAS	Scores (ordinal scale)	At the end of each session
Dentist's satisfaction and stress	10 cm VAS	Scores (ordinal scale)	At the end of each session
Child pain perceived by the dentist	10 cm VAS	Scores (ordinal scale)	At the end of each session
Child pain/distress according to observational scale	Observational scale "The Faces, Legs, Activity, Cry and Consolability" Pain Assessment Tool, Brazilian version—FLACC [19]	Score (ordinal scale)	At the end of the first treatment session (evaluation performed using the digital recording of the session)
Adverse events for sedated participants	Research instrument *Tracking and Reporting Outcomes of Procedural Sedation* TROOPS [20]	Frequency	During sedation and recovery
Unfavorable signs (protective stabilization)	Specific form	Frequency	Shortly after the dental procedure
The longevity of composite resin and glass ionomer cement restorations	Criteria for evaluation of occlusal [21] or occlusoproximal restorations [22]	Frequency (grouped categories in success/failure)	After the intervention, follow-up sessions of 4, 8, and 12 months
Child physiological stress	Salivary cortisol concentration, in mg/dL	Variation in cortisol levels during dental treatment (area under the curve)	Per session, at pre-determined moments: at arrival at the treatment session, 25 min after the end of the procedure

Selected observers from both sites will be trained to assess children's behavior (OSUBRS) and pain/distress (FLACC) by collectively discussing the theory behind each tool and applying the scales to 10 videos from similar participants undergoing dental treatment recorded for previous studies. Then, observers will independently watch rounds of diverse five videos to score children's behavior and pain until we obtain a minimal inter-rater agreement (intraclass correlation coefficient) of 0.7. Having received a satisfactory inter-rater agreement, observers will score this trial’s videos (one video by one observer). Every two weeks, observers will analyze one in every five videos they evaluated before estimating the intra-rater agreement coefficient.

Additionally, this study proposes an economic evaluation made from the payer's perspective (Brazilian National Health System), measuring the effect on children's behavior during the dental procedure according to the VAS assessed by the operator. An effectiveness analysis will compare the two behavioral management strategies according to the different available variables by measuring the costs (inputs) and consequences (results) of the interventions. An incremental cost-effectiveness analysis will be performed to classify the two strategies and verify the target population's health benefits [24, 25]. Direct costs for the two groups and professionals involved in the service will be identified, measured, and valued. We will not include the values for dental treatment performed, as these are included in the dentist's consultation time. Patient costs with transportation and time will not be analyzed. Each intervention's total cost will be estimated from official price lists regulated by the Ministry of Health.

### Participants recruitment and timeline

**Fig. 2:**
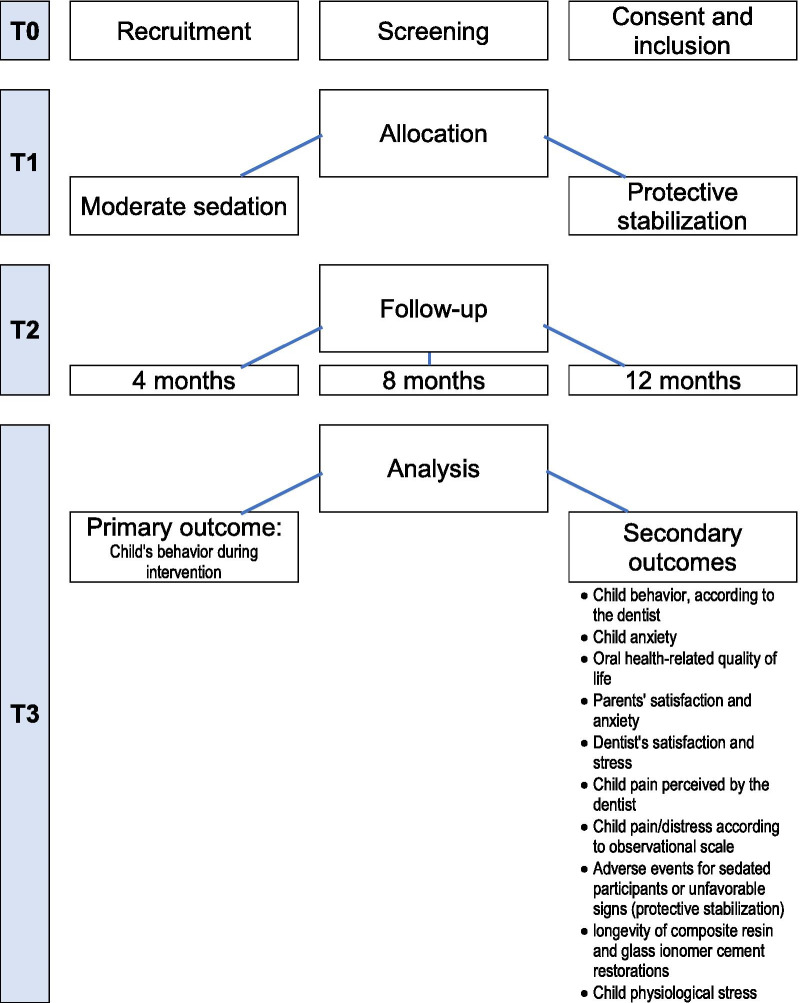
Flow diagram of the clinical trial's phases

Recruitment was initially planned to occur from January 2020 to December 2021 but has been suspended during the national emergency proclamation concerning the Novel Coronavirus Disease (COVID-19) outbreak since March 2020. We anticipate resuming recruitment in August 2021. After allocation and treatment in one of the groups, these will be followed up for 12 months. The detailed timeline for data collection is summarized in Fig. 2.

In this prospective study, the participant will follow a systematic sequence of steps to collect data for the proposed outcomes (Table 2).

**Table 2:**
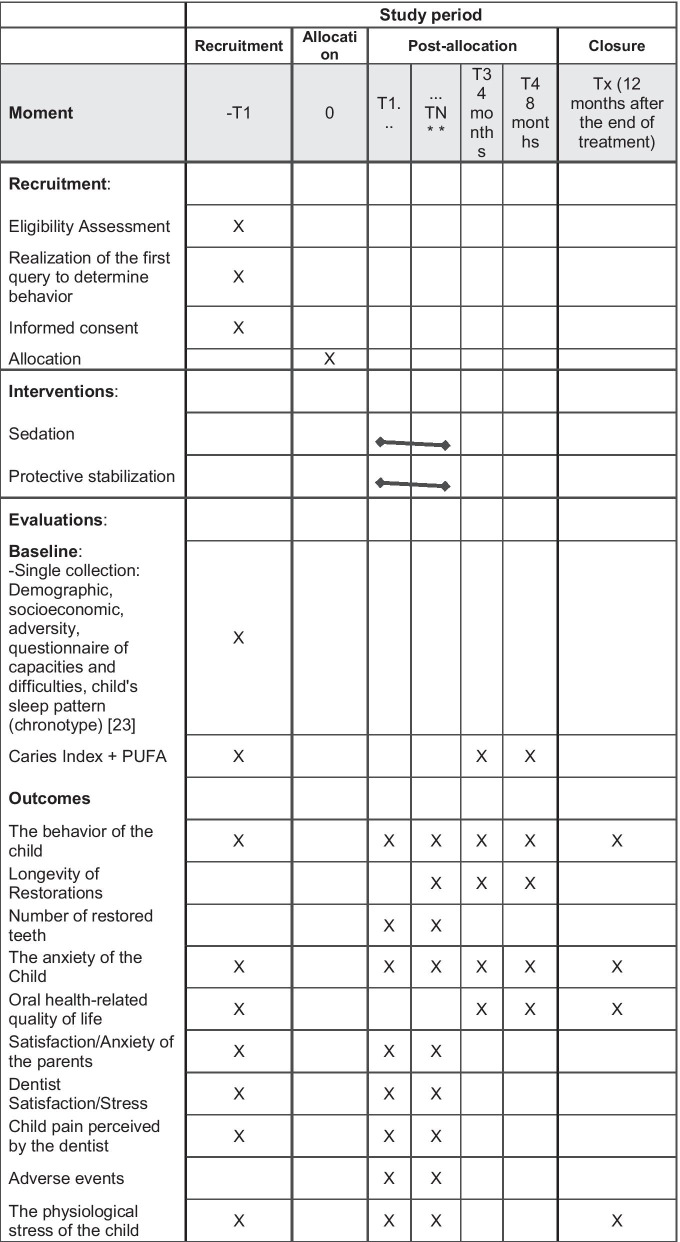
Schedule of inclusion and data collection for each participant, according to SPIRIT 2013

^*^The allocation is non-randomized; Sedation for UFG or protective stabilization for USP

^**^Dental treatment under sedation or protective stabilization will be performed in as many sessions as are necessary to complete the procedures

### Sample size

Preliminary estimates indicate that, based on adults prescribed an anxiolytic, the minimum clinically relevant difference in the VAS scale is between 10 and 15 mm [26]. This study would require a sample size of 63 for each group to achieve a power of 80% and a significance level of 5% (one-tailed) to detect an actual difference average between the intervention groups of 15 mm (assuming a grouped standard deviation of 30 units). To avoid significant losses in the final sample size, after 12 months of follow-up, we will add 20% to each group, which implies 76 children/group, i.e., a total sample size of 152 children.

According to age, the estimated sample will be distributed in 38 children aged 2–3 years and 38 children of 4–6 years in each center to perform subgroup analysis.

The sample size will be recalculated when the minimum number of 30 cases in each center is reached, based on the values observed in this study's conditions (interim analysis).

### Data analysis

#### Statistical methods

Descriptive and bivariate analyses will be performed in the statistical software PrismaGraphPad and IBM SPSS, considering the significance level of 5%.

Data summaries (frequencies, means/standard deviation, or medians/quartiles) will be calculated, as appropriate, for demographic and socioeconomic, psychosocial, medical and dental history, strengths and difficulties questionnaire, and oral health-related quality of life measures.

Based on all children who attend the first intervention consultation, an intention-to-treat analysis will be used for the outcomes “percentage of quiet behavior (OSUBRS) during treatment”, “parents’ satisfaction”, and “operators’ satisfaction”, using the Student's t-test or Mann–Whitney as appropriate. We will define the value “zero” for OSUBRS when children have their first intervention consultation aborted (no completion of the planned dental procedure). Adverse events (sedation group) or unfavorable restraint signs (protective stabilization) will be described. In this stage, the control of missing data is not foreseen because if a participant does not attend the first intervention consultation, she/he will be excluded from the study. Subgroup analysis will be performed for ages, considering the primary outcome.

Secondary analyses will include, but are not limited to:

- Children's pain during dental procedures performed under sedation or protective stabilization (Student's t-test or Mann–Whitney test);
- Children’s behavior and anxiety progress throughout treatment and follow-up consultations (Cox regression);
- Impact on oral health-related quality of life, comparing the scores obtained in the follow-up sessions with those of baseline (Paired T-Test or Wilcoxon in pairs of moments and ANOVA or Friedman to assess more than two evaluation moments);
- Longevity of glass ionomer cement and composite resin restorations (Kaplan–Meier survival analysis with Log-rank test);

For the secondary analyses, cases of withdrawal (children do not attend the follow-up sessions) will be included by appropriate methods of the imputation of lost data.

A ratio or quotient will express the cost-effectiveness analysis results. The numerator is the cost and the denominator the effectiveness, i.e., costs per unit of effectiveness, in our study ‘quiet behavior'. The incremental cost-effectiveness ratio (ICER) will be calculated by dividing the mean difference in the cost of sedation of a child compared to the protective stabilization due to the difference in the median probability of success between the two groups.

## Discussion

The United Nation's convention on children's rights underpins each child's entitlement to be treated safely and with dignity. In dentistry, this is especially important when behavioral management techniques are needed to manage those who have fear/anxiety or challenging behavior.

There has been a decline in protective stabilization by American pediatric dentists [27] in favor of pharmacological management, such as sedation [28]. Non-specialist dentists in Norway understand that the use of restraint, although an ethical dilemma in pediatric dentistry, might be acceptable when it is integral to a conscious sedation procedure [29]. However, protective stabilization alone is still considered necessary for several countries because of poorly developed access to sedation services; this is a particular dilemma for dentists treating children from underprivileged socioeconomic backgrounds. Indeed, Brazilian caregivers from this socioeconomic group were more likely to accept physical protective stabilization a second time for their children who had cognitive impairment [30]. In such underprivileged contexts, active protective stabilization provokes emotional discomfort in dentists, mothers, and psychologists but is tolerated because it allows the children's dental treatment [31]. More research on children’s challenging behavior management is needed to inform pediatric dental services in Brazil and other countries with similar contexts.

To the best of our knowledge, this is the first clinical trial to evaluate and compare the effectiveness of moderate sedation with protective stabilization for children with fear/anxiety or dental behavior problems needing dental care. Cost-effectiveness will also be assessed as a secondary outcome to inform sedation dental health service development in Brazil.

This clinical trial will be case-matched rather than randomized due to sedation being available only in the UFG center, a place with infrastructure, equipment, and professionals trained and qualified to develop ambulatory sedation. Similarly, as the interventions are quite different, they will not be possible to blind. In each center, multiple operators will provide the interventions; therefore, they will be trained to the needed routine to minimize service differences between operators.

The primary outcome (children's behavior according to OSUBRS) will be assessed by trained, calibrated observers with a methodology that has been proved helpful in a previous investigation [32]. The OSUBRS is a tool that offers a precise measurement of the child's behavior during dental care but is not feasible in the day-to-day clinic, as it requires a lot of effort for application because it depends on videos and specific software [33]. Additionally, we are assessing children's behavior according to a VAS reported by the dentist. VAS was used to estimate the sample size in this study preliminarily because we do not have OSUBRS estimates in non-sedated children. Most importantly, it is an adequate measure to quickly assess children's dental anxiety during dental treatment from the operator's perspective [34].

We will investigate outcomes related to the patient's (child/family) and the practitioners’ views, thus complying with the recommendations from the Cochrane systematic review on pediatric dental sedation [4]. Moreover, we propose to measure children's anxiety and behavior both at baseline and at follow-up, providing further strength through longitudinal data [4]. Compared to other studies, we will avoid carry-over bias since a crossover design will not be employed [4, 35].

Thus, our study wishes to answer whether sedated children have better behavior during dental treatment than those undergoing protective stabilization. If this hypothesis is supported, this non-randomized clinical trial will offer new knowledge in child dental health care and provide evidence to inform service development both in Brazil and worldwide.

### Trial status

The trial has been recruiting participants since January 2020 but is now suspended because of the COVID-19 outbreak. The end of the recruitment phase is planned for December 2021.

## Supplementary Information

**Additional file 1**. SPIRIT checklist.

**Additional file 2**. Details of clinical and laboratory procedures.

## Data Availability

The datasets used or analyzed during the current study will be available from the corresponding author on reasonable request.

## Abbreviations

B-ECOHIS: Brazilian version of Early Childhood Oral Health Impact Scale
CEDACORE: Children Experiencing Dental Anxiety—Collaboration on Research and Education
CHOOSE: Clinical holding or/and sedation
DBMP: Dental behavior management problem
DFA: Dental fear/anxiety
FIS: Facial Image Scale
FLACC: "The Faces, Legs, Activity, Cry and Consolability" Pain Assessment Tool
FOUSP: University of São Paulo School of Dentistry
HVGIC: High-viscosity glass ionomer cement
NESO: (UFG dental sedation center “Núcleo de Estudos em Sedação Odontológica”)
SPIRIT: Standard Protocol Items for Clinical Trials
SUS: Brazilian National Health System
UFG: Federal University of Goiás (“Universidade Federal de Goiás”)
VAS: Visual Analogue Scale
