# Supplementary material for: Sedation versus protective stabilization for dental treatment of children with caries and challenging behavior at the dentist (CHOOSE): a study protocol for a non-randomized clinical trial
Source: BMC Oral Health. 2021 May 12;21:256. doi: 10.1186/s12903-021-01594-0 (PMC8115863; doi:10.1186/s12903-021-01594-0)
Supplement: Supplementary file 2 — Additional file 2. Details of clinical and laboratory procedures. [file 12903_2021_1594_MOESM2_ESM.docx]

Additional File 2 – Clinical and laboratory procedures

1. **Dental examination appointment**

The first consultation consists of screening, medical examination (center UFG), and the child’s dental examination. The parents/legal guardians will be informed about the research as soon as the inclusion criteria are met. The consent process may take more than one session, if necessary. Shortly after the informed consent is signed, the caregiver will respond to specific forms and questionnaires to provide the investigated data as below.

- 1. Demographic, socioeconomic data and biological:
     1. Child: age, sex, attendance in daycare center/school/preschool, chronotype.
        1. Chronotype evaluated by Circadian Energy Scale (CIRENS) [1]: Classifies a person as morning, night, or intermediate type. This instrument consists of three questions about the usual child’s energy level (very low, low, moderate, high, very high) answered by the caregiver, being categorized from 1 to 5 in the morning, afternoon, and night. The sum of each period’s values is considered the child’s total energy and varies from 3 to 15. The child’s chronotype (i.e., morning, night, or intermediate type) is derived by subtracting the morning value from the night value. The CIRENS chronotype varies between -4 and +4, where the child is considered morning type if the result is ≤ -2, intermediate type if between -1 and +1, and night type if ≥+2.
     2. Caregiver: sex, age, degree of kinship with the child.
     3. Family: maternal schooling, core components, income.
  2. Psychosocial data:
     1. Adverse childhood experiences: Exposure of the child to adverse conditions such as divorce, parental incarceration, domestic violence, drug and alcohol abuse, mental illness, and emotional, physical or sexual abuse [2].
     2. Strengths and Difficulties Questionnaire (SDQ-Por), version with impact for parents of children aged 2-4 years and 4-17 years [3,4]: Aim to evaluate children’s mental health. It consists of 25 items (10 on strengths, 14 on difficulties, and one neutral). The SDQ is divided into five subscales, each with five assertions (emotional symptoms, conduct problems, hyperactivity, relationship problems with colleagues, and pro-social behavior). The answer options are “false” (zero points), “more or less true” (one point), or “true” (two points), totaling 0 to 40 points; The cutoff score for the total difficulty score is 17 for the parents’ version.
     3. Early Childhood Oral Health Impact Scale (B-ECOHIS) [5]. This instrument has 13 questions divided into two main sections: The impact section of the child (part one) with nine questions, and the family impact section (part two), with four questions. Each of the 13 questions is evaluated using a five-point gradual scale with the following response options: “Never” (0); “Rarely” (1); “Sometimes” (2); “Often” (3); “Very often” (4) and “I Do not Know” (5). The scores of each question, except the item “Do not know”, “are added to obtain the instrument’s total score. This score can range from zero to 52, and the highest scores indicate a more negative impact on the quality of life related to oral health.
     4. Facial Image Scale (FIS) [6]: Children aged three years and above will be asked to self-report their dental anxiety using FIS. First, they will be explained about the scale according to their cognitive development and then asked to pick one of the tool’s six faces.
  3. Medical and dental assessment
     1. Standardized dental exams will be performed in both centers to allow diagnosis and treatment planning.
     2. Medical examination: In the UFG Center, children will undergo a medical exam to assess their systemic condition and obtain vital signs. The sedation procedure will follow the American Academy of Pediatrics and American Academy of Pediatric Dentistry [7] recommendations to maximize the benefits and reduce the child’s risks. It will be planned for the following appointment.

1. **Intervention appointment**

All consultations with sedation will be scheduled for the morning period and will follow steps to standardize the procedures in both centers as much as possible.

- 1. Baseline saliva collection: Once the child arrives at the dental clinic reception, a research assistant will collect the child’s saliva using the Salivette tube (Sarstedt Inc., Nümbrecht, Germany). A cotton roll will be positioned in the child’s mouth for a period of one to two minutes until soaking with the saliva. Subsequently, this cotton roll will be relocated in the tube stored in a thermal box containing ice until centrifugation.
  2. Sedative administration: In the center UFG, children will receive medications as planned and wait for the initial drug effect.
  3. Dental procedure: In the first dental rehabilitation appointment, the child will preferably receive a restoration of composite resin after local anesthesia and rubber dam isolation or glass ionomer cement using the atraumatic restorative treatment technique. At least one dental restoration will be completed, even if the child presents negative/definitely negative behavior; the goal is to perform as many procedures as possible (child with positive behavior). Active protective stabilization may be used if necessary. An operator and respective assistant will provide the tooth restoration. A pulse oximeter will be connected to the child’s finger to allow the heart rate and oxygen saturation. The child’s caregiver will be together in the operatory during the whole appointment.
     1. In the center UFG, a trained observer will be included in the team to monitor the child’s systemic conditions from the administration of the sedative to discharge. After the dental procedure ending, the child should stay in the recovery room to meet the discharge criteria.
     2. In both centers, the first restorative consultation will be filmed for further evaluation of the behavior and pain felt by the child during dental procedures. The assessments will be conducted later by trained and calibrated researchers and using the observational tools Ohio State University Behavioral Scale (OSUBRS) [8] and Faces, Legs, Activity, Cry and Consolability (FLACC) Pain Assessment Tool [9].
     3. There will be another saliva collection during the procedure and 25 minutes after the end of the appointment as item 2.1, as cortisol reaches its peak in saliva around 20-30 minutes after the stressful stimulus.
     4. Soon after the completion of the dental restorative procedure:
        1. The pediatric dentist should register, independently:
           1. Tooth restoration quality and the number of restored teeth: The width and depth of marginal defects, surface wear, and excess or lack of material will be measured using the WHO periodontal probe CPI, which has a spherical tip of 0.5 mm in diameter.
           2. According to a 10 cm Visual Analogue Scale (VAS), perception of the child’s behavior scored for nearest millimeter.
           3. Perception of the child’s pain in the moments of local anesthesia, removal of the carious tissue, and insertion of the restorative material using the VAS.
           4. Satisfaction with the procedure using VAS.
           5. Self-report stress during the procedure using VAS.
        2. Reports from caregiver and child:
           1. Caregiver: satisfaction and anxiety with the intervention using VAS.
           2. Child self-report anxiety (FIS) [6] with this appointment according to item 1.2.4.
  4. For the sedated children, any intra-operative and post-operative (up to 24 hours) adverse events will be noted according to the tool Tracking and Reporting Outcomes of Procedural Sedation TROOPS [10]. Children that receive protective stabilization will be evaluated for the occurrence of unfavorable signs as marks on the skin.

1. **Video’s assessment**

Trained and calibrated observers will assess children’s behavior and pain during the procedure by watching the videos produced during the intervention appointment. The behavior will be evaluated continuously and independently according to the OSUBRS [8], using the software Observer XT (Noldus, The Netherlands) and explained in the manuscript body.

To assess pain, one of the observers should analyze the videos using the FLACC Pain Assessment Tool [9]. The FLACC scale consists of five categories (F = face, L = legs, A = activity, C = crying, C = consolability) with scores ranging from zero to two, indicating the intensity of the child’s pain according to the observed behavior. The score of each category should be attributed at the end of the video evaluation, opting for the score representing the worst condition experienced by the child. In the end, the scores of the categories will be summed, obtaining a total score for the child’s pain varying from 0 (no pain) to 10 (severe pain). Each video will be analyzed twice by the same observer: 1- videos will be watched without the audio to evaluate the categories “faces, legs and activity”; 2- videos will be watched keeping the audio, for the assessment of “crying and consolability”.

1. **Salivary cortisol analysis**

Salivary cortisol levels will be measured to indicate the child’s physiological stress. The saliva samples will be centrifuged at 4500 rpm for 15 minutes and subsequently stored in Eppendorf and frozen tubes in the freezer-800 (SANYO/VIP® PLUSTm, USA) until the time of analysis.

The laboratory analysis to measure cortisol in the saliva samples collected will be Held in the Saliva Analysis Laboratory of the UFG School of Dentistry, using an enzyme immunoassay (ELISA) and an immunosorbent enzymatic Kit (Salimetrics Kit, LLC, USA) using the competitive ELISA to measure salivary cortisol. All steps in this method will follow the information that accompanies the same Kit. The wells will be analyzed in a photometer (DNM-9602 Microplate Reader, California USA) with an absorbance of 450 nm. The cortisol level will be determined according to the standard curves, prepared according to the manufacturer (Salimetrics Kit, LLC, USA), and with a detection limit of 0.012 mcg/dL to 3.0 mcg/dL.

**References**

[1] Ottoni GL, Antoniolli E, Lara DR. The Circadian Energy Scale (CIRENS): two simple questions for a reliable chronotype measurement based on energy. Chronobiol Int. 2011 Apr;28(3):229-37.

[2] Crouch E, Radcliff E, Nelson J, Strompolis M, Martin A. The experience of adverse childhood experiences and dental care in childhood. Community Dent Oral Epidemiol. 2018 Oct;46(5):442-448.

[3] Goodman R. The Strengths and Difficulties Questionnaire: a research note. J Child Psychol Psychiatry. 1997 Jul;38(5):581-6.

[4] Cury CR, Golfeto JH. Strengths and difficulties questionnaire (SDQ): a study of school children in Ribeirão Preto. Braz J Psychiatry. 2003 Sep;25(3):139-45.

[5] Scarpelli AC, Oliveira BH, Tesch FC, Leo AT, Pordeus IA, Paiva SM. Psychometric properties of the Brazilian version of the Early Childhood Oral Health Impact Scale (B-ECOHIS). BMC Oral Health. Jun 2011 13; 11:19.

[6] Buchanan H, Niven N. Validation of a Facial Image Scale to assess child dental anxiety. Int J Paediatr Dent. 2002 Jan;12(1):47-52.

[7] Coté CJ, Wilson S; AMERICAN ACADEMY OF PEDIATRICS; AMERICAN ACADEMY OF PEDIATRIC DENTISTRY. Guidelines for Monitoring and Management of Pediatric Patients Before, During, and After Sedation for Diagnostic and Therapeutic Procedures. Pediatrics. 2019 Jun;143(6):e20191000.

[8] Lochary ME, Wilson S, Griffen AL, Coury DL. Temperament as a predictor of behavior for conscious sedation in dentistry. Pediatr Dent. 1993 Sep-Oct;15(5):348-52.

[9] Silva FC, Thuler LC. Cross-cultural adaptation and translation of two pain assessment tools in children and adolescents. J Pediatr (Rio J). 2008;84(4):344-349.

[10] Roback MG, Green SM, Andolfatto G, Leroy PL, Mason KP. Tracking and Reporting Outcomes of Procedural Sedation (TROOPS): Standardized Quality Improvement and Research Tools from the International Committee for the Advancement of Procedural Sedation. Br J Anaesth. 2018 Jan;120(1):164-172.
